# Frozen embryo transfer in the menstrual cycle after moderate-severe ovarian hyperstimulation syndrome: a retrospective analysis

**DOI:** 10.1186/s12884-022-05239-0

**Published:** 2022-12-06

**Authors:** Yulu Yang, Damin Zhu, Qiushuang Wang, Cong Ma, Danyang Li, Jing Wang, Ping Zhou, Zhaolian Wei, Xiaoqing Peng, Yunxia Cao, Xiaofeng Xu

**Affiliations:** 1 Reproductive Medicine Center, Department of Obstetrics and Gynecology, the First Affiliated Hospital of Anhui Medical University, No 218 Jixi Road, Hefei, 230022 Anhui China; 2 NHC Key Laboratory of Study on Abnormal Gametes and Reproductive Tract (Anhui Medical University), No 81 Meishan Road, Hefei, 230032 Anhui China; 3 Key Laboratory of Population Health Across Life Cycle (Anhui Medical University), Ministry of Education of the People’s Republic of China, No 81 Meishan Road, Hefei, 230032 Anhui China; 4 Department of Obstetrics and Gynecology, the First Affiliated Hospital of Anhui Medical University, Hefei, 230022 Anhui China; 5 Anhui Provincial Engineering Research Center of Biopreservation and Artificial Organs, No 81 Meishan Road, Hefei, 230032 Anhui China; 6 Anhui Province Key Laboratory of Reproductive Health and Genetics, No 81 Meishan Road, Hefei, 230032 Anhui China; 7 Anhui Provincial Institute of Translational Medicine, No 81 Meishan Road, Hefei, 230032 Anhui China

**Keywords:** OHSS, Frozen embryo transfer, Maternal outcomes, Pregnancy complication, Neonatal outcome

## Abstract

**Background:**

Ovarian hyperstimulation syndrome (OHSS) is a rare but serious complication of controlled ovarian stimulation. Frozen-embryo transfer (ET) is prompted to be performed in the next menstrual cycles after cancellation of fresh-ET after occurrence of OHSS. However, effects of frozen-ET in the second menstrual cycle have never been investigated. Therefore, this study aimed to assess this in the menstrual cycle after OHSS.

**Methods:**

The OHSS group included 342 women with moderate-severe OHSS who underwent the first frozen-ET in the second menstrual cycle in the First Affiliated Hospital of Anhui Medical University from June 2018 to September 2019. A total of 342 women without OHSS who received frozen-ET in the second menstrual cycle were selected as control group matched by age, body mass index, fertility history, ovulation induction scheme. Uni- and multi-variable conditional logistic regression was used to estimate the association between moderate-severe OHSS and pregnancy outcomes.

**Results:**

There were no significant differences in maternal outcomes (miscarriage, preterm birth and pregnancy complications including gestational diabetes mellitus, pregnancy-induced hypertension, placenta previa, premature rupture of membranes and postpartum hemorrhage) and in neonatal outcome (birth-weight and body length, neonatal congenital diseases and other complications) between the two groups in either uni- or multi-variable models.

**Conclusions:**

Frozen-ET in the menstrual cycle after OHSS has similar maternal and neonatal outcomes as in women without OHSS. This study indicates that frozen-ET could be performed in the second menstrual cycle in women who recovered from moderate-severe OHSS.

**Supplementary Information:**

The online version contains supplementary material available at 10.1186/s12884-022-05239-0.

## Background

Ovarian hyperstimulation syndrome (OHSS) is a complication associated with controlled ovarian stimulation (COS) during infertility treatment [1–3]. Moderate-severe OHSS occurs approximately in 1-5% of in vitro fertilization (IVF) cycles [4–6], whilst mild OHSS is usually unnoticed. OHSS is more severe in China with moderate stage in 3-6% in the Chinese society and severe in 0.2-1% among women undergoing IVF [7, 8]. Moderate OHSS clinical manifestations include abdominal pain, ovarian enlargement with cyst formation, ascites or pleural effusion, and severe cases can be life-threatening [9–11]. Studies have found increased odds of multiple pregnancies, gestational diabetes mellitus (GDM), preterm birth and pregnancy-induced hypertension (PIH) among OHSS women compared with women without OHSS [12–15].

Frozen embryo transfer (frozen-ET) has been shown to reduce the risk of severe OHSS and has been prompted to avoid deterioration of OHSS [14, 16–18]. When moderate or severe OHSS is occurring, fresh-ET cycles are cancelled to allow the women to recover from OHSS in clinics [5, 19, 20]. Termination of fresh-ET is frustrating to both infertile couples and clinicians, and earlier embryos transfer is helpful to releave the unhealthy psychology. Although attempts of frozen-ET in the menstrual cycle after embryo retrieval are usually performed among women with OHSS, pregnancy outcomes are unknown. Therefore, this study aimed to assess pregnancy outcomes of frozen-ET in the menstrual cycle after oocytes retrieval among women with moderate-severe OHSS.

## Methods

### Study population

From the 10.090 women in the Reproductive Center of the First Affiliated Hospital of Anhui Medical University 5.746 women had the first frozen cycle during the period from June 2018 to September 2019. Among them, 705 (12.3%) had moderate-severe OHSS. Inclusion criteria included women ≤40 years who were at high risk of OHSS [21, 22] and frozen-ET in the menstrual cycle after oocytes retrieval. Exclusion criteria were: (1) fresh-ET cycle, (2) refrozen-ET cycle, (3) donor sperm and donor oocyte, (4) preimplantation genetic testing, (5) chromosomal abnormality, (6) clinical data missing, and (7) other diseases that may affect pregnancy outcomes, including diabetes, hypothyroidism, hyperthyroidism, hyperprolactinemia, pituitary tumor, systemic lupus erythematosus, rheumatoid, rheumatic cardiopathy, sicca syndrome and mental diseases.

**Fig. 1:**
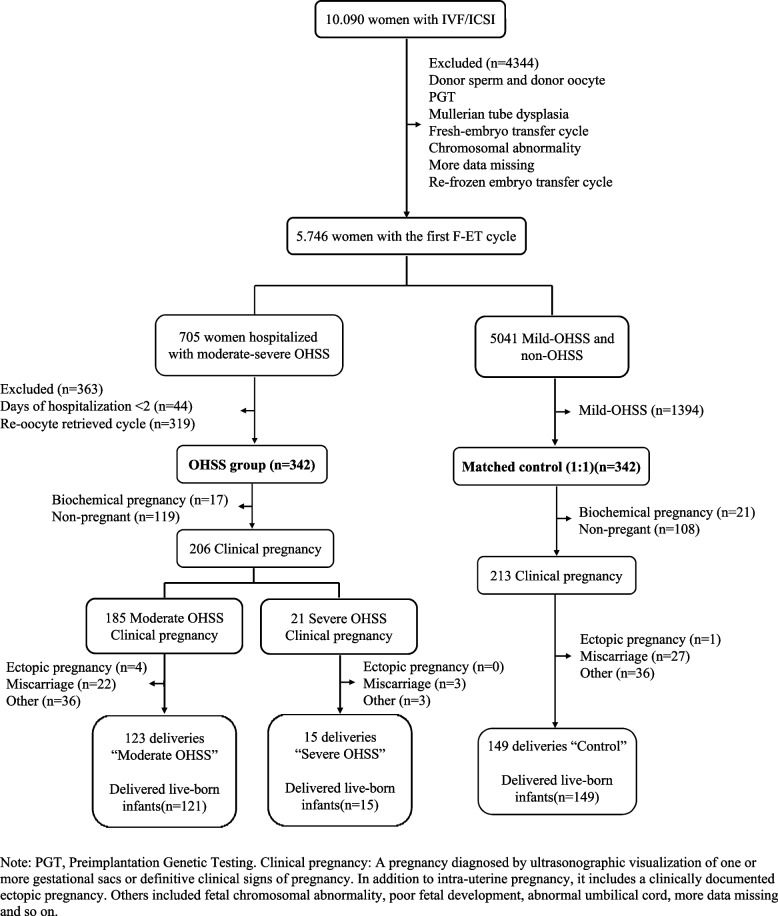
Flow chart

Inclusion criteria were fulfilled in 342 (48.5%) women allocated to the OHSS group. The control group consisted of 342 women, who conceived via the first Frozen-ET cycle during the same time period and did not develop OHSS, matched by age, body mass index (BMI), etiology, fertility history and ovulation induction scheme. The flowchart of the study is summarized in Fig. 1.

### Classification of OHSS

Moderate and severe OHSS was classified according to the American Society for Reproductive Medicine criteria [23]. Moderate OHSS is defined as symptoms including a considerable degree of abdominal discomfort, plus nausea, vomiting, and/or diarrhoea, ovaries usually 5 to 12 cm in diameter. Classification of severe OHSS was as follows: ovaries larger than 12 cm in diameter, marked gastrointestinal symptoms, oliguria, ascites, pleural effusion, hypovolemia and hemoconcentration with electrolyte imbalance. Information used to categorize OHSS included size of the ovaries on the day of oocyte collection, ascites, pleural effusion and laboratory data (e.g. Hct, electrolyte, liver enzymes).

### Pregnancy outcomes

Data on pregnancy outcomes related to mother and neonate were extracted from the Electronic Medical Records. Maternal outcomes included biochemical pregnancy, clinical pregnancy, ectopic pregnancy, early pregnancy, abortion rate, successful childbirth and pregnancy complications rate (e.g. GDM, PIH, placenta previa, premature rupture of membranes and postpartum hemorrhage). Neonatal outcomes included gestational age at birth, birth-weight and body length, congenital defects rate, jaundice rate and neonatal mortality. Serum human chorionic gonadotropin (HCG) levels on the 14^th^ day after ET were assessed to determine biochemical pregnancy. Clinical pregnancy was defined as a sac visible by ultrasonography with a fetal heartbeat at 7 weeks after ET. Miscarriage with positive heart activity was used to define loss of clinical pregnancy.

### Other variables

Demographic and clinical data of the women were retrieved from the Electronic Medical Records including age, years of infertility, types of infertility, number of pregnancies, causes of infertility, BMI, basic sex hormone levels (follicle-stimulating hormone, estradiol_2_, prolactin, luteinising hormone, testosterone and total dosage of gonadotropin). On trigger day, the following data were extracted: number of follicles, retrieved oocytes, transplantable embryos, high-quality embryos and sex hormone values (estradiol_2_, luteinising hormone and progesterone). The information of implantation included transfer interval, endometrial thickness on transfer day, number of transferred embryos, clinical pregnancy rate and ectopic pregnancy rate.

### Statistical analysis

Continuous data were presented as mean ± standard deviation and count data were described using frequencies and percentages. Normality tests of continuous data were performed by visual inspection of the histogram and distribution curve. Comparison of continuous variables between the groups was conducted using Student’s t-test and analysis of variance. Chi-squared or Fisher exact test were used for comparison of categorical variables as appropriate. Multivariable regression was used to determine the association of moderate-severe OHSS with maternal and neonatal outcomes after controlling for confounders. Potential confounders with *P-values* < 0.20 in the univariate analysis were included in the multivariate model. Confounders in the multivariable models included BMI, basal serum follicle-stimulating hormone, estradiol_2_, prolactin, luteinising hormone, total dosage of gonadotropin, total days of gonadotropin, number of follicles, serum estradiol_2_, luteinising hormone, progesterone on trigger day, number of retrieved oocytes, number of transplantable embryos, number of high-quality embryos. *P-values* < 0.05 indicated statistically significant differences. Statistical analyses were conducted using SPSS (Windows version 24.0, IBM-SPSS, Chicago, IL).

## Results

### Women’ characteristics

Baseline characteristics of the moderate-severe OHSS and matched control groups are presented in Table 1. The incidence of severe OHSS was 111/5746 (1.9%) per IVF cycle. Follicle-stimulating hormone level was lower and luteinising hormone higher in the OHSS group as compared to controls. There were no significant differences between the two groups of each 342 women in the ovulation induction program. Total amount of gonadotropins used in the OHSS group was lower (*P* < 0.05). On trigger day, serum levels of estradiol_2_ and progesterone were significantly higher and the number of removed oocytes and transplantable embryos were significantly greater in the OHSS group than in the control group (*P* < 0.05). Regarding perinatal outcomes, 123/304 (40.5%) successful births in the moderate OHSS group, 15/38 (39.5%) in the severe group and 149/342 (43.6%) in the control group (*P* = 0.478; Fig. 1).

**Table 1:** Basic data and transplant characteristics of women with OHSS and without OHSS

Characteristic	OHSS Group (*n* = 342)	Matched Control (*n* = 342)	*P* value
Maternal age (y)	28.32 ± 3.55	28.32 ± 3.55	1.000
Paternal age (y)	29.83 ± 4.13	29.71 ± 4.26	0.695
BMI (kg/m^2^)	22.33 ± 3.07	22.82 ± 2.43	0.051
Duration of infertility (y)	3.01 ± 1.95	3.18 ± 2.01	0.256
Types of infertility (%, n)			1.000
Primary infertility	66.08 (226/342)	66.08 (226/342)	
Secondary infertility	33.92 (116/342)	33.92 (116/342)	
Number of pregnancies	0.54 ± 0.91	0.60 ± 1.04	0.433
Indication of infertility (%, n)			0.952
Tubal factor	31.58 (108/342)	29.53 (101/342)	
PCOS	34.80 (119/342)	35.67 (122/342)	
Male factor	28.07 (96/342)	28.95 (99/342)	
Other	5.56 (19/342)	5.85 (20/342)	
Basal serum sex hormone levels			
FSH (pmol/L)	6.18 ± 1.53	6.93 ± 1.82	< 0.001
E_2_ (pmol/L)	161.50 ± 89.14	176.24 ± 133.20	0.089
PRL (pmol/L)	25.54 ± 65.47	18.70 ± 31.87	0.083
LH (pmol/L)	7.75 ± 5.34	6.97 ± 5.23	0.054
T (pmol/L)	3.55 ± 10.74	2.92 ± 8.26	0.387
Iatrogenic ovarian stimulation (%, n)			0.860
Long protocol (Long term effect)	57.02 (195/342)	55.97 (188/342)	
Antagonist protocol	40.25 (138/342)	42.40 (145/342)	
Other	2.63 (9/342)	2.63 (9/342)	
Total Gn dosage (U)	1849.46 ± 561.80	1996.26 ± 735.41	*0.003
Total days of Gn (day)	10.72 ± 1.83	10.98 ± 2.35	0.106
Number of follicles			
Total follicles	23.26 ± 7.34	15.49 ± 7.11	*0.000
Left ovary	10.95 ± 4.00	7.63 ± 4.81	*0.000
Right ovary	12.32 ± 4.61	7.86 ± 3.56	*0.000
Trigger day			
Endometrial thickness (mm)	11.78 ± 2.46	11.82 ± 2.42	0.830
E_2_ (pmol/L)	17,850.03 ± 9588.84	14,313.96 ± 4400.77	*< 0.001
LH (pmol/L)	2.66 ± 3.08	3.04 ± 3.39	0.164
P (pmol/L)	6.38 ± 3.13	4.24 ± 2.66	*< 0.001
Number of retrieved oocytes	25.47 ± 6.38	15.37 ± 6.88	*< 0.001
Number of transplantable embryos	9.65 ± 4.32	6.32 ± 3.70	*< 0.001
Number of high-quality embryos	8.46 ± 4.25	5.54 ± 3.54	*< 0.001
Transplant interval (day)	95.77 ± 43.55	92.93 ± 48.39	0.419
Number of transferred embryos	1.32 ± 0.47	1.31 ± 0.46	0.805

Qualitative data are n (%); quantitative data are mean ± SD

*BMI* body mass index, *PCOS* polycystic ovary syndrome, *FSH* follicle-stimulating hormone, *E2* estradiol_2_, *PRL* prolactin, *LH* Luteinising hormone, *T* Testosterone, *Gn* Gonadotropins, *P* Progesterone

* Significant difference

### Pregnancy outcomes between moderate-severe OHSS group and control group

Rates of biochemical pregnancy, clinical pregnancy, extrauterine pregnancy and early abortion were similar in both groups (Table 2). Rates of GDM, PIH, placenta previa, premature rupture of membranes and postpartum hemorrhage were all comparable between two groups (all *P* > 0.05). Similarly, gestational age at birth, preterm birth rate or caesarean section rate in OHSS group were not statistically different from matched controls. Moreover, none of the maternal and neonatal outcomes were associated with moderate-severe OHSS in either univariable or multivariable regression models.

**Table 2:** Pregnancy outcomes of women with moderate-severe OHSS and controls

Characteristic	OHSS Group (*n* = 342)	Matched Control (*n* = 342)	c*OR*	a*OR*
			(95% CI)	(95% CI)
Biochemical pregnancy rate (%, n)	65.2 (223/342)	68.4 (234/342)	0.95 (0.69-1.30)	0.82 (0.56-1.20)
Clinical pregnancy rate (%, n)	60.2 (206/342)	62.3 (213/342)	0.97 (0.71-1.32)	0.78 (0.54-1.14)
Ectopic pregnancy rate (%, n)	1.9 (4/206)	0.5 (1/213)	5.05 (0.65-45.45)	9.26 (0.50-170.81)
Miscarriage rate (%, n)	12.4 (25/202)	12.7 (27/212)	0.91 (0.51-1.61)	0.99 (0.51-1.96)
Childbirth	138	149	0.90 (0.66-1.22)	0.73 (0.51-1.04)
Pregnancy complication				
GDM (%, n)	0.7 (1/138)	2.0 (3/149)	0.62 (0.15-2.51)	0.84 (0.14-4.91)
PIH (%, n)	3.6 (5/138)	3.4 (5/149)	1.25 (0.36-4.37)	1.35 (0.32-5.75)
Placenta previa (%, n)	1.5 (2/138)	1.3 (2/149)	1.25 (0.18-8.94)	202.4 (0.28-146,434.89)
Premature rupture of membranes (%, n)	3.6 (5/138)	2.0 (3/149)	2.10 (0.50-8.86)	1.35 (0.27-6.76)
Postpartum hemorrhage (%, n)	0.7 (1/138)	0 (0/149)	5,331,600.14	3.71
Preterm birth (%, n)	19.6 (27/138)	14.1 (21/149)	1.16 (0.64-2.11)	1.01 (0.51-2.00)
Cesarean section (%, n)	67.4 (93/138)	68.5 (102/149)	1.03 (0.66-1.60)	0.67 (0.40-1.13)
Gestational age (weeks)	37.9 ± 2.0	38.2 ± 1.7	0.26(−0.18-0.71)	3.03 (−0.28-6.34)
Birth weight (g)	3313.5 ± 565.2	3391.7 ± 574.7	108.03(−31.38-247.45)	153.90 (− 120.13-427.93)
Birth length (cm)	50.1 ± 2.5	50.0 ± 2.7	0.30(−0.28-0.87)	3.37 (−0.57-7.32)
Neonatal congenital diseases (%, n)	0.7 (1/138)	0.7 (1/149)	1.25 (0.08-20.08)	6.93
Neonatal death (%, n)	1.5 (2/138)	0 (0/149)	1.07	9.64
Other neonatal complication (%, n)	2.2 (3/138)	2.0 (3/149)	1.25 (0.25-6.25)	1.87 (0.24-14.80)

Qualitative data are n (%); quantitative data are mean ± SD

*CI* confidence interval, *GDM* gestational diabetes mellitus, *PIH* gestational hypertension, *cOR* crude odds ratio under univariable, *aOR* adjusted odds ratio under multivariable, *CI* confidence interval. Multivariable was adjusted for BMI, basal serum follicle-stimulating hormone, basal serum estradiol_2_, basal serum prolactin, basal serum luteinising hormone, total gonadotropin dosage, total days of gonadotropin, number of follicles, serum estradiol_2_ level on trigger day, serum luteinizing hormone, serum progesterone on trigger day, number of retrieved oocytes, number of transplantable embryos, number of high-quality embryos

### Pregnancy outcomes among moderate OHSS group, severe OHSS group and controls

Comparisons of women’ characteristics among the moderate OHSS, severe OHSS and control group are shown in Supplementary Table 1. Women with moderate OHSS had the lowest level of basal follicle-stimulating hormone level and the difference was statistically significant compared with controls. The number of follicles was the highest in women with moderate OHSS compared with severe OHSS and controls (*P* < 0.05). Additionally, numbers of both left and right ovary follicles in severe OHSS were higher than in controls (*P* < 0.001). Estradiol_2_ and progesterone levels on trigger day in moderate OHSS were significantly higher than those in the control group (*P* < 0.001). Hormone levels in severe OHSS were higher than that in control group, however, lower than in moderate OHSS. The number of transplantable and high-quality embryos in severe OHSS was highest compared with the other groups. Supplementary Table 2 summarized pregnancy data for moderate to severe OHSS and controls. Furthermore, there were no significate differences in the odds of pregnancy outcomes among moderate OHSS, severe OHSS and non-OHSS women (Table 3).

**Table 3:** Pregnancy outcomes of women with moderate-severe OHSS and controls

Characteristic	Severe vs Moderate	Moderate vs Control	Severe vs Control
	*OR* (95% CI)	*OR* (95% CI)	*OR* (95% CI)
Biochemical pregnancy rate (%, n)	0.69 (0.34-1.41)	0.73 (0.49-1.10)	0.51 (0.25-1.04)
Clinical pregnancy rate (%, n)	0.82 (0.41-1.66)	0.72 (0.49-1.07)	0.60 (0.29-1.21)
Ectopic pregnancy rate (%, n)	*0.00	7.61 (0.39-147.40)	*0.00
Early pregnancy abortion rate (%, n)	0.65 (0.15-2.95)	0.93 (0.46-1.87)	0.61 (0.13-2.77)
Childbirth	1.08 (0.53-2.20)	0.70 (0.48-1.02)	0.75 (0.37-1.54)
Pregnancy complication			
GDM (%, n)	*0.00	0.72 (0.12-4.37)	*0.00
PIH (%, n)	*0.00	1.19 (0.28-5.15)	*0.00
Placenta previa (%, n)	*0.00	201.40 (0.30-145,378.49)	*0.00
Premature rupture of membranes (%, n)	*0.00	1.15 (0.22-5.93)	*0.00
Postpartum hemorrhage	*0.00	6.65	2.06
Preterm birth (%, n)	1.23 (0.34-4.54)	1.05 (0.52-2.14)	1.30 (0.35-4.82)
Cesarean section (%, n)	0.86 (0.28-2.67)	0.61 (0.36-1.06)	0.53 (0.17-1.64)
Neonatal congenital diseases (%, n)	*0.00	3.67	*0.00
Neonatal death (%, n)	*0.00	8.91	0.70
Other neonatal complication (%, n)	*0.00	1.71 (0.21-13.85)	*0.00

Qualitative data are n (%); quantitative data are mean ± SD

*CI* confidence interval, *GDM* gestational diabetes mellitus, *PIH* gestational hypertension, *OR* odds ratio, *CI* confidence interval. Multivariable was adjusted for BMI, basal serum follicle-stimulating hormone, basal serum estradiol2, basal serum prolactin, basal serum luteinising hormone, total gonadotropin dosage, total days of gonadotropin, number of follicles, serum estradiol_2_ level on trigger day, serum luteinising hormone, serum progesterone on trigger day, number of retrieved oocytes, number of transplantable embryos, number of high-quality embryos

* Significant difference

## Discussion

To the best of our knowledge, this is the first study investigating the effect of frozen-ET in the menstrual cycle after oocyte retrieval on pregnancy outcomes among women with OHSS. We found that there are no differences in the odds of pregnancy outcomes between frozen-ET among women with moderate-severe OHSS compared with controls, and this persisted among women with moderate OHSS, severe OHSS and controls. These findings indicate that frozen-ET in the subsequent menstrual cycle after OHSS has comparable pregnancy outcomes with women without OHSS regardless of the severity of OHSS.

HCG used in COS stimulates the secretion of vascular endothelial growth factor (VEGF). Over-secretion of VEGF, however, increases the capillary permeability resulting in the clinical symptoms of OHSS related to systemic vascular endothelial dysfunction and micro-thromboembolism in GDM and PIH [24–30]. Treatment strategy is clinically adopted to remit the acute condition, and fresh-ET is prompted to be cancelled. Anticipated conception propels the earliest frozen-ET in the menstrual cycle after OHSS, but the therapeutic effect has never been reported. This study has good homogeneity and comparability, indicated by the indifference in most clinical characteristics between women with and without OHSS. The proportion of OHSS in this study is slightly higher than that in general infertile populations [23, 31]. This could be attributed by the high proportion of women with polycystic ovaries (PCO) (4713/11090, 42.5%) in our reproductive center [32]. The number of retrieved oocytes, number of transplantable embryos and high-quality embryos were higher in the OHSS group, which is in line with previous findings due to hyper-responsiveness of ovarian tissue [33, 34]. The total dosage of gonadotropin for ovarian stimulation was significantly lower in women with OHSS than in those without OHSS, which is expected because women with OHSS tends to be more sensitive to ovarian stimulation [35]. Moreover, levels of estradiol_2_ on trigger day in both severe and moderate OHSS groups were higher than controls, which is consistent with the theory that circulating high estradiol_2_ levels could be of predictive value for the occurrence of OHSS [36, 37].

Fresh-ET in women with OHSS has been shown to increase the rate of loss of pregnancy, preterm birth, GDM and PIH compared with women conceived via IVF and did not develop OHSS, whilst we did not find difference in the odds of pregnancy outcomes between women with OHSS and without OHSS [38–40]. This suggests the superiority of frozen-ET to fresh-ET for women with OHSS. This could be because the increase of VEGF, IL-1α, IL-6 and TGF-β triggered by COS has been back to physiological level in approximately 20 days after OHSS [36, 41–44]. This suggests that the pathological consequences of OHSS are eliminated in the subsequent menstrual cycle. Moreover, elimination of the detrimental effects of OHSS on pregnancy outcomes was regardless of the severity of OHSS.

## Conclusions

This study found no differences in pregnancy outcomes of the first frozen-ET cycle between women with moderate-severe OHSS and those without OHSS. This suggests that once women are recovered from OHSS in the next menstrual cycle, frozen-ET could be performed for anticipated conception. This study provides supportive evidence for the clinical treatment strategy of performing frozen-ET in the menstrual cycle after OHSS.

## Supplementary Information

**Additional file 1: Supplementary Table 1.** Basic data of moderate-severe OHSS group and matched Control

**Additional file 2: Supplementary Table 2.** Ovulation induction and transplant information of moderate-severe OHSS and control.

## Data Availability

The datasets used and/or analysed during the current study are available from the corresponding author on reasonable request.

## Abbreviations

OHSS: Ovarian Hyperstimulation Syndrome
F-ET: Frozen Embryo Transfer
IVF: In-Vitro Fertilization
COS: Controlled Ovarian Stimulation
HCG: Human Chorionic Gonadotropin
VEGF: Vascular Endothelial Growth Factor
GDM: Gestational Diabetes Mellitus
PIH: Pregnancy-Induced Hypertension
BMI: Body Mass Index
