# Supplementary material for: Frozen embryo transfer in the menstrual cycle after moderate-severe ovarian hyperstimulation syndrome: a retrospective analysis
Source: BMC Pregnancy Childbirth. 2022 Dec 6;22:907. doi: 10.1186/s12884-022-05239-0 (PMC9724267; doi:10.1186/s12884-022-05239-0)
Supplement: Supplementary file 1 — Additional file 1: Supplementary Table 1. Basic data of moderate-severe OHSS group and matched Control [file 12884_2022_5239_MOESM1_ESM.docx]

**Supplementary table 1**. Basic data of moderate-severe OHSS group and matched Control

| Characteristic | Moderate Group  (n=304) | Severe Group  (n=38) | Control  (n=342) | *P* value  1-2 | *P* value  1-3 | *P* value  2-3 |
| --- | --- | --- | --- | --- | --- | --- |
| Maternal age (y) | 28.38±3.54 | 27.76±3.66 | 28.32±3.55 | 0.309 | 0.805 | 0.363 |
| Paternal age (y) | 29.90±4.13 | 29.32±4.13 | 29.71±4.26 | 0.420 | 0.565 | 0.585 |
| BMI (kg/m^2^) | 22.38±3.10 | 21.96±2.76 | 22.82±3.43 | 0.462 | 0.086 | 0.126 |
| Duration of infertility (y) | 3.04±1.98 | 2.74±1.75 | 3.18±2.01 | 0.376 | 0.375 | 0.193 |
| Types of infertility (%, n) |  |  |  | 0.076 | 0.668 | 0.109 |
| Primary infertility | 64.48(196/304) | 78.95(30/38) | 66.08(226/342) |  |  |  |
| Secondary infertility | 35.52(108/304) | 21.05(8/38) | 33.92(116/342) |  |  |  |
| Number of pregnancies | 0.56±0.91 | 0.39±0.89 | 0.60±1.04 | 0.317 | 0.604 | 0.213 |
| Indication of infertility (%, n) |  |  |  | 0.857 | 0.970 | 0.823 |
| Tubal factor | 31.25(95/304) | 34.21(13/38)  101 | 29.53(101/342) |  |  |  |
| PCOS | 34.87(106/304) | 34.21(13/38) | 35.67(122/342) |  |  |  |
| Male factor | 27.96(85/304) | 28.95(11/38) | 28.95(99/342) |  |  |  |
| Other~~*~~ | 5.92(18/304) | 2.63(1/38) | 5.85(20/342) |  |  |  |
| Basal serum sex hormone levels |  |  |  |  |  |  |
| FSH (pmol/L) | 6.15±1.50 | 6.46±1.78 | 6.93±1.82 | 0.280 | ^*^0.000 | 0.102 |
| E_2_ (pmol/L) | 161.69±88.42 | 159.90±95.89 | 176.24±133.20 | 0.927 | 0.104 | 0.400 |
| PRL (pmol/L) | 27.13±69.27 | 12.81±5.05 | 18.70±31.87 | 0.106 | 0.038 | 0.503 |
| LH (pmol/L) | 7.57±5.10 | 9.15±6.88 | 6.97±5.23 | 0.082 | 0.146 | 0.016 |
| T (pmol/L) | 3.28±10.46 | 5.79±12.68 | 2.92±8.26 | 0.127 | 0.638 | 0.080 |
| Iatrogenic ovarian stimulation |  |  |  | 0.446 | 0.901 | 0.458 |
| Long protocol (Long term effect) | 56.58(172/304) | 60.53(23/38) | 54.97(188/342) |  |  |  |
| Antagonist protocol | 41.12(125/304) | 34.21(13/38) | 42.40(145/342) |  |  |  |
| Other | 2.30(7/304) | 5.26(2/38) | 2.63(9/342) |  |  |  |
| Total Gn dosage (U) | 1855.27±565.71 | 1802.97±534.39 | 1996.26±735.41 | 0.643 | *0.006 | 0.085 |
| Total days of Gn (day) | 10.72±1.85 | 10.68±1.69 | 10.98±2.35 | 0.920 | 0.123 | 0.417 |
| Number of follicles |  |  |  |  |  |  |
| Total follicles | 23.63±7.39 | 20.26±6.20 | 15.49±7.11 | *0.007 | *0.000 | *0.000 |
| Left ovary | 11.17±4.06 | 9.13±2.99 | 7.63±4.81 | *0.007 | *0.000 | 0.047 |
| Right ovary | 12.47±4.66 | 11.13±4.13 | 7.86±3.56 | 0.06 | *0.000 | *0.000 |
| Trigger day |  |  |  |  |  |  |
| Endometrial thickness (mm) | 11.74±2.42 | 12.06±2.79 | 11.82±2.42 | 0.451 | 0.696 | 0.563 |
| E_2_ (pmol/L) | 17985.47±10151.85 | 16850.15±3060.14 | 14313.96±4400.77 | 0.403 | *0.000 | 0.059 |
| LH (pmol/L) | 2.53±2.91 | 3.61±4.03 | 3.04±3.39 | 0.07 | 0.07 | 0.332 |
| P (pmol/L) | 6.40±3.14 | 6.22±3.09 | 4.24±2.66 | 0.736 | *0.000 | *0.000 |
| Number of retrieved oocytes | 25.68±6.35 | 23.76±6.43 | 15.37±6.88 | 0.092 | *0.000 | *0.000 |
| Number of transplantable embryos | 9.58±4.18 | 10.21±5.37 | 6.32±3.70 | 0.362 | *0.000 | *0.000 |
| Number of high-quality embryos | 8.37±4.10 | 9.18±5.33 | 5.54±3.54 | 0.366 | *0.000 | *0.000 |
| Transplant interval (day) | 94.48±39.24 | 106.08±68.92 | 92.93±48.39 | 0.143 | 0.668 | 0.095 |
| Number of transferred embryos | 1.32±0.47 | 1.29±0.46 | 1.31±0.46 | 0.681 | 0.735 | 0.797 |

Note: Qualitative data are n (%); quantitative data are mean ± SD. *BMI*, body mass index; *PCOS*, polycystic ovary syndrome; *FSH*, follicle-stimulating hormone; *E*_2_, estradiol_2_; *PRL*, prolactin; *LH*, luteinising hormone; *T*, testosterone. 1-2 was moderate group vs. severe group; 1-3 value was moderate group vs. control; 2-3 value was severe group vs. control.

* Significant difference.
