# Supplementary material for: Frozen embryo transfer in the menstrual cycle after moderate-severe ovarian hyperstimulation syndrome: a retrospective analysis
Source: BMC Pregnancy Childbirth. 2022 Dec 6;22:907. doi: 10.1186/s12884-022-05239-0 (PMC9724267; doi:10.1186/s12884-022-05239-0)
Supplement: Supplementary file 2 — Additional file 2: Supplementary Table 2. Ovulation induction and transplant information of moderate-severe OHSS and control. [file 12884_2022_5239_MOESM2_ESM.docx]

**Supplementary table 2**. Ovulation induction and transplant information of moderate-severe OHSS and control.

| Characteristic | Moderate Group  (n=304) | Severe Group  (n=38) | Control  (n=342) | | *P* value  1-2 | *P* value  1-3 | *P* value  2-3 |
| --- | --- | --- | --- | --- | --- | --- | --- |
| Biochemical pregnancy rate (%, n) | 66.12 (201/304) | 57.89 (22/38) | 68.42 (234/342) | 0.316 | | 0.533 | 0.189 |
| Clinical pregnancy rate (%, n) | 60.86 (185/304) | 55.26 (21/38) | 62.28 (213/342) | 0.507 | | 0.710 | 0.399 |
| Ectopic pregnancy rate (%, n) | 2.16 (4/185) | 0 (0/21) | 0.47 (1/213) | 1.000 | | 0.188 | 1.000 |
| Early pregnancy abortion rate (%, n) | 11.89 (22/185) | 14.29 (3/21) | 12.74 (27/212) | 1.000 | | 0.799 | 1.000 |
| Delivery | 123 | 15 | 149 | 0.907 | | 0.425 | 0.629 |
| Pregnancy complication |  |  |  |  | |  |  |
| GDM (%, n) | 0.81 (1/123) | 0 (0/15) | 2.01 (3/149) | 1.000 | | 0.629 | 1.000 |
| PIH (%, n) | 3.25 (4/123) | 6.67 (1/15) | 3.36 (5/149) | 0.443 | | 1.000 | 0.443 |
| Placenta previa (%, n) | 1.63 (2/123) | 0 (0/15) | 1.34 (2/149) | 1.000 | | 1.000 | 1.000 |
| Premature rupture of membranes (%, n) | 3.25 (4/123) | 6.67 (1/15) | 2.01 (3/149) | 0.443 | | 0.705 | 0.321 |
| Postpartum hemorrhage | 0.81 (1/123) | 0 (0/15) | 0 (0/149) | 1.000 | | 0.452 | 1.000 |
| Preterm delivery (%, n) | 19.51 (24/123) | 20.00 (3/15) | 14.09 (21/149) | 1.000 | | 0.231 | 0.815 |
| Cesarean section (%, n) | 66.67 (82/123) | 73.33 (11/15) | 68.46 (102/149) | 0.8191 | | 0.754 | 0.923 |
| Duration of gestation (weeks) | 37.73±2.23 | 35.87±10.09 | 38.20±1.73 | 0.022 | | 0.193 | 0.004 |
| Neonatal weight (g) | 3274.72±628.65 | 3401.33±594.09 | 3391.68±574.67 | 0.441 | | 0.110 | 0.953 |
| Neonatal length (cm) | 49.57±2.92 | 50.13±3.60 | 50.02±2.66 | 0.466 | | 0.191 | 0.883 |
| Neonatal congenital diseases (%, n) | 0.81 (1/123) | 0 (0/15) | 0.67 (1/149) | 1.000 | | 1.000 | 1.000 |
| Neonatal death (%, n) | 1.63 (2/123) | 0 (0/15) | 0 (0/149) | 1.000 | | 0.204 | 1.000 |
| Neonatal other complication (%, n) | 2.44 (3/123) | 0 (0/15) | 2.01 (3/149) | 1.000 | | 1.000 | 1.000 |

Note: qualitative data are n (%); quantitative data are mean ± SD. *GDM*, gestational diabetes mellitus; *PIH*, gestational hypertension. 1-2 was moderate group vs. severe group; 1-3 value was moderate group vs. control; 2-3 value was severe group vs. control.
